# Lower Richness of Small Wild Mammal Species and Chagas Disease Risk

**DOI:** 10.1371/journal.pntd.0001647

**Published:** 2012-05-15

**Authors:** Samanta Cristina das Chagas Xavier, André Luiz Rodrigues Roque, Valdirene dos Santos Lima, Kerla Joeline Lima Monteiro, Joel Carlos Rodrigues Otaviano, Luiz Felipe Coutinho Ferreira da Silva, Ana Maria Jansen

**Affiliations:** 1 Laboratory of Tripanosomatid Biology, Oswaldo Cruz Foundation (FIOCRUZ), Rio de Janeiro, Brazil; 2 Laboratory of Quantitative Methods, National School of Public Health Sérgio Arouca, Oswaldo Cruz Foundation (FIOCRUZ), Rio de Janeiro, Brazil; 3 Laboratory of Cartography, Military Institute of Engineering, Rio de Janeiro, Brazil; Universidad Autónoma de Yucatán, Mexico

## Abstract

A new epidemiological scenario involving the oral transmission of Chagas disease, mainly in the Amazon basin, requires innovative control measures. Geospatial analyses of the *Trypanosoma cruzi* transmission cycle in the wild mammals have been scarce. We applied interpolation and map algebra methods to evaluate mammalian fauna variables related to small wild mammals and the *T. cruzi* infection pattern in dogs to identify hotspot areas of transmission. We also evaluated the use of dogs as sentinels of epidemiological risk of Chagas disease. Dogs (n = 649) were examined by two parasitological and three distinct serological assays. kDNA amplification was performed in patent infections, although the infection was mainly sub-patent in dogs. The distribution of *T. cruzi* infection in dogs was not homogeneous, ranging from 11–89% in different localities. The interpolation method and map algebra were employed to test the associations between the lower richness in mammal species and the risk of exposure of dogs to *T. cruzi* infection. Geospatial analysis indicated that the reduction of the mammal fauna (richness and abundance) was associated with higher parasitemia in small wild mammals and higher exposure of dogs to infection. A Generalized Linear Model (GLM) demonstrated that species richness and positive hemocultures in wild mammals were associated with *T. cruzi* infection in dogs. Domestic canine infection rates differed significantly between areas with and without Chagas disease outbreaks (Chi-squared test). Geospatial analysis by interpolation and map algebra methods proved to be a powerful tool in the evaluation of areas of *T. cruzi* transmission. Dog infection was shown to not only be an efficient indicator of reduction of wild mammalian fauna richness but to also act as a signal for the presence of small wild mammals with high parasitemia. The lower richness of small mammal species is discussed as a risk factor for the re-emergence of Chagas disease.

## Author Summary

The classical methodology of mapping works with discrete units and sharp boundaries does not consider gradient transition areas. Spatial analysis by the interpolation method, followed by map algebra, is able to model the spatial distribution of biological phenomena and their distribution and eventual association with other parameters or variables, with a focus on enhancing the decision power of responsible authorities. Acute Chagas Disease outbreaks are increasing in the Amazon Basin as result of oral transmission. This scenario requires a new approach to identify hotspot transmission areas and implement control measures. We applied a geospatial approach using interpolation and map algebra methods to evaluate mammalian fauna variables related to these outbreaks. We constructed maps with mammalian fauna variables including the infection rates by *Trypanosoma cruzi*, in dogs and small wild mammals. The results obtained by visual examination of the maps were validated by statistical analysis. We observed that high prevalence of *T. cruzi* infection in dogs and small wild mammals was associated with mammal lower richness. Monitoring of *T. cruzi* infection in dogs may be a valuable tool for detecting the fauna lower richness of small wild mammals and elucidating the transmission cycle of *T. cruzi* in the wild.

## Introduction

The causative agent of Chagas disease, *Trypanosoma cruzi* (Chagas, 1909), is a multi-host parasite capable of infecting almost all tissues of more than one hundred mammal species [1]. Dozens of species of insects from the Triatominae subfamily can act as its vector. Except for the epimastigote form, all other *T. cruzi* evolutive forms can infect mammals by oral and congenital pathways as well as by contamination of the mucosae and skin abrasions by infected triatomine feces. The biological plasticity of *T. cruzi* results in transmission cycles that are characterized by being multivariate and complex on unique temporal and spatial scales [1], [2].

Classically, Chagas disease was characterized as prevalent in rural populations, where houses were heavily infested by domiciliated triatomine species, mainly *Triatoma infestans* (Klug, 1834). The campaigns launched by the “Cone Sul” Intergovernmental Commission to eliminate the domiciliary vectors succeeded in that Brazil and other countries in South America are currently considered free from domestic transmission of Chagas via *Triatoma infestans* [3]. However, extradomiciliary vectorial transmission, domiciliary or peridomestic transmission by non-domiciliated vectors and oral transmission by ingestion of food contaminated by feces from infected insects (the principal method of current transmission), pose new challenges. In fact, mainly in the northern part of the Brazil, the number of Chagas disease outbreaks due to the ingestion of food contaminated by infected triatomine feces are increasing [4]–[6]. This is currently considered a new epidemiological scenario, demanding systematic surveillance methods that consider all components of the transmission cycle as well as the landscape and ambient conditions in which transmission is occurring.

In several reports, the maintenance of biodiversity has been pointed to as a strong buffering system and regulator in the dispersal of parasites; this has been named the “Dilution Effect”. Such a dilution effect has already been demonstrated to be of importance in the transmission of West Nile encephalitis, Hantavirus, Lyme disease and Schistosomiasis [7], [8]. Despite the demonstration of this effect, studies on the impact of biodiversity variation on the *T. cruzi* transmission cycle in the wild mammals using a Geographical Information System (GIS) have been scarce up to now [9]–[12]. The destruction of an ecosystem imposes important area and food restrictions onto wild mammal populations and may promote their greater contact with humans. The consequence of this process is the increased opportunity for contact among humans, domestic animals and wildlife [2]. In this scenario, the transmission of *T. cruzi* may be increased due to the following: (i) positive selection of generalist species with high transmissibility competence such as *Didelphids* and some caviomorph rodents that undoubtedly adapt and survive in degraded habitats, (ii) the consequent amplification of the parasite's transmission cycle due to higher abundance of competent reservoir species and (iii) the increased prevalence of infected bugs. In addition, the scarcity of food sources for triatomines (i.e., loss of wildlife due to destruction of the environment) led them to invade human dwellings and annexes [13]. Also, the quantitative and spatial patterns of the landscape and artificial lighting in human dwellings play a fundamental role in domiciliary invasion [14]. Human residences acting as light-traps for insects has significant epidemiological importance, as species with high rates of *T. cruzi* infection are drawn to human dwellings [15]. In this scenario, peridomestic mammals are more frequently exposed. Thus, their infection usually precedes the human infection. Hence, dogs have been proposed as being suitable sentinel hosts for *T. cruzi* transmission in areas at risk for human infection [16], [17].

Dogs can be important reservoirs of this parasite. They display both a high prevalence of infection and high parasitemia as evidenced in Panama [17], Argentina [18], Venezuela [19], Mexico [20], and the United States [21]. In contrast, in Brazil, a high serum prevalence in dogs has also been described in several areas, but the importance of dogs as a reservoir species has been described as negligible because no high patent parasitemia has been observed in these animals [16], [22].

Geospatial analysis based on the fundamental concepts of landscape epidemiology [23] is a powerful tool in the study of the association between landscape- and vector-borne diseases such as Chagas disease, Schistosomiasis and American Visceral Leishmaniasis [9], [10], [24]. Geospatial analysis allows for the identification of disease risk areas and disease interactions with the environment [10], [24].

The classical methodology of mapping works with discrete units and sharp boundaries, and does not consider transition areas. Nevertheless, environmental and biological phenomena are typically continuous and exhibit a gradual transition from one characteristic to another. Unlike the classical methodology, spatial analysis by the interpolation method, followed by map algebra, is able to model the spatial distribution of the continuous biological phenomena, representing the distribution and association of these phenomena in a more realistic way. This modeling can enhance and facilitate decision making [25].

The present paper evaluates and compares *T. cruzi* infection rates of dogs from three Brazilian biomes, including areas where orally transmitted Chagas disease outbreaks were reported and areas where Chagas disease is endemic. Our objectives were to (i) assess the impacts of lower richness of small wild mammals on the prevalence of *T. cruzi* infection in dogs, (ii) discuss the role of dogs in the transmission cycle of *T. cruzi* and their putative role as sentinels and (iii) to assess the interpolation and map algebra method as a tool for the construction of potential Chagas disease risk area maps.

## Materials and Methods

### Study area

**Figure 1:**
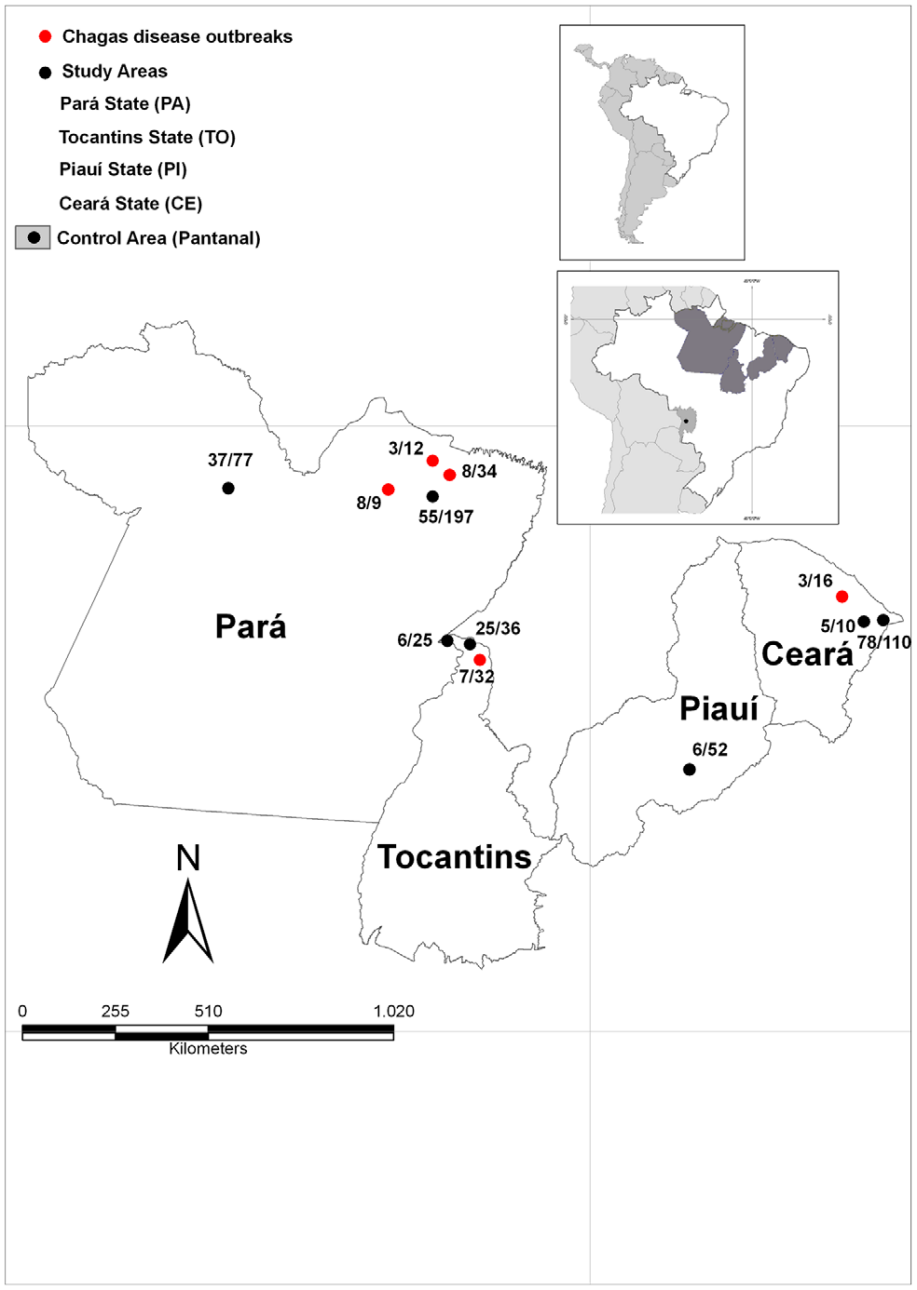
Geographical location of study area. Study areas are located in the 3 geographic Brazilian biomes: Amazon (Pará and Tocantins states), Caatinga (Piauí and Ceará states) and Pantanal (Mato Grosso do Sul state, control area). **Red markers** indicate areas investigated immediately after the occurrence of Chagas disease outbreaks; **Black markers** indicate non-outbreak areas.

Dogs (n = 649) were sampled in 3 geographic Brazilian biomes: the Amazon, Caatinga and Pantanal, from 5 states and 13 municipalities (28 localities) (Figure 1 and Table 1).

**Table 1:** Seroprevalence of *Trypanosoma cruzi* infection in dogs from three biomes: Caatinga, Amazon Forest and Pantanal.

Biome/State	Municipalities	Localities	Coordinates		Serological+/total (%)
**Caatinga**	Jaguaruana	Caatinguinha	04°48′28″S	37°47′09″W	11/26 (42)
Ceará		Córrego das Melancias	04°50′10″S	37°47′20″W	22/23 (96)
		Dió	04°50′23″S	37°46′19″W	19/29 (65)
		Perímetro Irrigado	04°49′18″S	37°50′22″W	18/23 (78)
		Figueiredo do Bruno	04°47′45″S	37°49′19″W	8/9 (89)
					**78/110 (71)**
	Redenção* 2	Salobro	04°11′48″S	38°42′56″W	1/2 (50)
		Alto Cassiano	04°13′18″S	38°43′56″W	1/9 (11)
		Manoel Dias	04°10′54″S	38°43′45″W	1/2 (50)
		Sítio Outeiro	04°12′21″S	38°44′09″W	0/3
					**3/16 (19)**
	Russas	Cipó	04°57′47″S	38°09′18″W	**5/10 (50)**
Piauí	João Costa^1^	Urban Area	08°33′24″S	42°26′12″W	**6/52 (11)**
**Amazon Forest**					
Tocantins	Augustinópolis	2000	05°24′11″S	48°01′09″W	11/12 (92)
		São Roque	05°30′26″S	48°02′46″W	14/24 (58)
					**25/36 (69)**
	Esperantina	São Francisco	05°23′32″S	48°28′46″W	**6/25 (24)**
	Axixá do Tocantins*	Lagoa de São Salvador	05°38′37″S	47°44′19″W	4/10 (40)
		Piquizeiro	05°42′09″S	47°44′29″W	3/22 (14)
					**7/32 (22)**
Pará	Abaetetuba	Ajuaí**	01°45′29″S	49°03′25″W	6/18 (33)
		Genipauba	01°45′29″S	48°54′01″W	0/4
		Panacaueira	01°48′18″S	49°06′15″W	11/26 (42)
		Urban Area	01°42′58″S	48°51′30″W	38/149 (25)
					**55/197 (28)**
	Belém*	Jurunas	01°28′14″S	48°30′10″W	2/15 (13)
		Val de Cans	01°23′12″S	48°28′17″W	6/19 (32)
					**8/34 (23)**
	Cachoeira do Arari* 2	Aranaí	01°05′42″S	48°39′39″W	2/4 (50)
		Furinho	01°05′14″S	48°39′07″W	0/2
		Mata Fome	01°04′15″S	48°37′50″W	1/5 (20)
		Sede Furo Grande	01°05′31″S	48°39′14″W	0/1
					**3/12 (25)**
	Curralinho*	São José da Povoação**	01°40′28″S	50°08′41″W	**8/9 (89)**
	Monte Alegre	Setor 11***	01°38′20″S	54°14′32″W	**37/77 (48)**
**Pantanal (Control Area)**					
Mato Grosso do Sul	Corumbá	Farms	19°34′29″S	56°14′44″W	**0/39**
					**241/649 (37)**

Footnotes

*: Chagas disease outbreaks.

**: Positive hemoculture.

***: Trypomastigote forms in fresh blood preparations.

1 -Data published in [22].

2 -Data published in [2].

Among these municipalities, samples were taken immediately after an outbreak of Acute Chagas Disease (ACD) from Redenção, Cachoeiro do Arari, Belém, Curralinho and Axixá do Tocantins. The other monitored areas were Abaetetuba, Monte Alegre, Augustinópolis, Esperantina, Jaguaruana, Russas and João Costa, while Corumbá (Midwest Brazil) was used as a control area. The areas included in our study reflect the locations where our laboratory has been developing research and reference services over the past few years.

### Biome Caatinga

The states of Piauí and Ceará display similar patterns: a high density of naturally *T. cruzi* infected Triatominae, which are the main vectors of disease in both regions. Despite that, no new vectorial transmission Chagas disease cases have been observed in the last decade and, as far as we know, only one outbreak has occurred due to oral transmission [2], [26].

The municipalities of Jaguaruana, Redenção and Russas, which are endemic for Chagas disease, are located in the mesoregion of lower Jaguaribe, in the northeastern state of Ceará. In Jaguaruana, the average annual temperature ranges from 23°C to 33°C. The collection area consists of clay and sandy soil plains, which are characterized as Caatinga, and include the typical vegetation of semi-arid areas. Redenção (ACD outbreak in 2006) is located in the Baturité mountain range region. The climate is semi-arid, and the average annual temperature ranges from 24°C to 35°C. The collection area, originally part of a tropical semi-humid forest, is currently characterized by secondary vegetation consisting of small trees (up to 6 m), rocky formations, and remnant patches of semi-humid forest near deforested areas occupied by monoculture plantations or unplanned households (slums). The municipality of Russas is located in the state of Ceará. The climate is semi-arid with average temperatures ranging from 18.8°C to 35.4°C. The vegetation comprise open scrub and savanna, with deciduous thorny forest areas. The municipality of João Costa is located in the southeast of the state of Piauí and is characterized as a megathermic semi-arid region. The average annual temperature ranges from 12°C to 39°C. The vegetation in this area displays the typical Caatinga features and residual semi-deciduous forest patches.

### Amazon biome

*T. cruzi* oral transmission in the Brazilian Amazon region has been reported since 1968 [5], [27], although this region was considered an endemic area for many years. Just after 2005, when the prevalence of Chagas cases in other parts of the country decreased and surveillance in the Amazonian region was improved, microepidemics of ACD began to appear regularly and frequently, mainly associated with the consumption of the palm-tree fruit açaí and other foods [4]–[6].

The municipalities of Abatetetuba and Belém (ACD outbreak in 2009) are located in the northeastern mesoregion of the state of Pará. Cachoeiro do Arari (ACD outbreak in 2006) and Curralinho (ACD outbreak in 2009) are located in the mesoregion of Marajó, while Monte Alegre is located in the lower Amazon mesoregion of Pará. The common climate is characterized as tropical humid, with regular rainfall and winds, and temperatures between 27°C and 36°C. The area is known as varzean, which is a freshwater swamp forest. In most of the collection areas, the original native vegetation (Amazonian forest) is being replaced by an extensive açaí fruit monoculture with a few remaining patches of the original vegetation at the river banks. The municipalities of Augustinópolis, Axixá do Tocantins (ACD outbreak in 2009) and Esperantina are located in the northwestern mesoregion of the Tocantins state, almost at the border of Pará. The climate of these cities is tropical subhumid, with maximum temperatures occurring during the dry season that reach 39°C.

### Pantanal biome (control area)

This region presents an enzootic cycle of *T. cruzi* transmission; however, it is not considered an endemic area for Chagas disease, as human cases have never been recorded in the region. This region comprises a large natural environment with a multiplicity of habitats and a wide variety of biodiversity. Farms encompass an area located in the core of a biodiversity corridor in the Pantanal of Mato Grosso do Sul, Brazil.

### Wild mammals

The capture of small wild and synanthropic mammals was performed as follows: live traps were arranged in linear transects, and the capture points were established with Tomahawk (Tomahawk Live Traps, Tomahawk, WI) and Sherman (H. B. Sherman Traps, Tallahassee, FL) traps. The traps were baited with a mixture of peanut butter, banana, oat and bacon and set at 20-m intervals in all types of vegetation formations and habitats. The trapped animals were taken to a field laboratory ≤2 km from the capture point, where the remaining procedures were performed. The trapped animals were examined for the prevalence and pattern of *T. cruzi* infection, as previously described [2], [9], [28], [29]. Some data (from the municipalities of João Costa, Cachoeira do Arari, Redenção) used in this meta-analysis comprise both already published studies [2], [9], [22] and some unpublished data (from the municipalities of Jaguaruana, Russas, Abaetetuba, Belém, Monte Alegre, Curralinho, Axixá do Tocantins, Augustinópolis and Esperantina) were collected by our laboratory. The sampling efforts to capture mammals were similar in all 28 localities (820-1.100 traps/night), with 4 or 5 nights of capture each season, and the captures were performed in every season of the year (Table S1 and Figure S1).

### Selection of dogs

The active search for dogs was conducted in the houses neighboring the linear transects where the small wild and synanthropic mammals were captured and in the houses where oral outbreaks of Chagas disease had occurred. Blood samples were collected from 649 dogs living in houses located in twelve municipalities from four Brazilian states. Dog blood was collected in three biomes; collections from the Caatinga (n = 188) and Amazon (n = 422) biomes were compared to collections from the Pantanal biome (n = 39) (Table 1).

### Parasitological and serological surveys

Herein, we considered that each dog represents one single event, even when related to the same house. This is due to: (i) dogs are separated individuals, differing each other in age, behavior, activities, etc… This fact is reflected in their different degrees of *T. cruzi* exposition; (ii) dogs have no pack behavior; and (iii) dogs are not confined in the intradomociliar area and have different and multiple opportunities to be infected during their activities. The interpretation of our results was based on different patterns of infection of the mammals. Fresh blood smears and hemoculture, when positive (especially the first due to its lower sensibility), show a high parasite load in the peripheral blood of the animals, which means a high chance of transmission to the vector, reflecting transmissibility. These tests are very specific but less sensitive – their importance lies in detecting infected animals that may represent a source of infection for the vector. Serological assays indicate infection of the animal. Therefore, serological positive and parasitological negative tests for a given animal demonstrates its infection with a low rate of parasite, this mammal is a host of the parasite, but is not involved in the amplification of parasite populations, i.e., its transmission potential to the vector is low.

Blood was collected from dogs in heparinized vacutainer tubes by puncture of the cephalic vein. To evaluate *T. cruzi* infection, four tests were conducted. Two of these tests were parasitological assays including (i) the examination of fresh blood smears (microscopic analysis) and (ii) a hemoculture assay, in which 0.2–0.4 mL of blood was cultured in two tubes containing Novy-Mc Neal-Nicole medium [NNN] with a liver infusion tryptose medium [LIT] overlay. When those tests were positive, the parasites were amplified for cryopreservation and DNA extraction for molecular characterization and two serologic diagnostic assays were performed: (iii) the Indirect Immunofluorescence Antibody Test (IFAT) as previously described [30] and (iv) the Enzyme-Linked Immunosorbent Assay (ELISA, Bio-Manguinhos, FIOCRUZ, Rio de Janeiro, RJ, Brazil). Disease diagnosis was based on serology by the ELISA (Cut-off: optical absorbance ≥0.200, mean±3 SD) and IFAT (Cut-off: titer of 1/40). Each microtiter polystyrene plate had 2 positive and 2 negative control sera. Animals were defined as seropositive when samples were reactive in both the IFAT and ELISA. Seropositive animals that displayed negative results in the parasitological assays were considered to have sub-patent infections. To evaluate possible cross-reactions and/or mixed infection by *T. cruzi* and *Leishmania* spp., dog sera were also assayed for *Leishmania infantum* using IFAT and the Rapid Test for Diagnosis of Canine Visceral Leishmaniasis (CVL) (TR DPP®, Bio-Manguinhos, FIOCRUZ, Rio de Janeiro, RJ, Brazil). The IFAT cut-off value adopted for *T. cruzi* infection was 1/40 when the IFAT result for *L. infantum* was lower than 1/40 and the DPP results were negative. When infected by *L. infantum*, dogs were considered positive for *T. cruzi* infection only when the IFAT titer was 1/80 or higher. For *L. infantum* infection, the adopted IFAT cut-off value was 1/40 when the infection was also confirmed by DPP and 1/80 when the DPP assay was negative. The interpretation of these tests in assemblage indicates the role played by the tested mammals in the transmission cycle.

### PCR amplification

DNA was extracted from logarithmic phase cultures and serum samples of dogs with patent parasitemia (positive blood slide smears) in the absence of hemocultivated parasites, using a phenol chloroform protocol [31]. PCR was performed using the primer pair S35 (5′-AAATAATGTACGGGGGAGATGCATGA-3′) and S36 (5′-GGGTTCGATTGGGGTTGGTGT-3′) [31]. Cyclic amplifications were performed with an initial denaturation of five minutes at 94°C, followed by 35 amplification cycles (94°C for 30 seconds, 55°C for 30 seconds, 72°C for 30 seconds) and a final ten-minute elongation step at 72°C. Each 25 µL total reaction volume contained 25 ng total DNA, 10 ρmol of each primer, 0.4 mM dNTPs, 2 mM MgCl_2_, and 2.5 U Taq polymerase (AmpliTaq®Gold, Applied Biosystems). PCR products were visualized on a 2% agarose gel after ethidium bromide staining. PCR resulted in a 330-bp amplicon for *T. cruzi* and a 760-bp amplicon and heterogeneous set of fragments ranging in size from 300 to 450 bp for *T. rangeli*.

### Geospatial analysis

The base map was acquired from the IBGE (Brazilian Institute of Geography and Statistics). The coordinates of all biological data were captured using a hand-held GPS (Global Positioning System) receiver (Garmim III GPS, Garmin International, Olathe, KS, USA) and recorded in the WGS 84 Datum (World Geodetic System 1984) geodetic coordinate system.

Maps representing the spatial distribution of *T. cruzi* infected dogs (response variable) and species richness, abundance and parasitological and serological prevalence of wild small mammals (covariables) were generated using the interpolation method of Inverse Distance Weighted (IDW) with the 12 nearest sampled data points selected. However, for map analysis, only the polygon of the studied municipality, (*i.e.*, only a local analysis) was used. A variable radius was applied specifying the number of nearest input sample points (n = 12) to perform interpolation. After that, we used map algebra to find evidence of spatial correlation of the response variable with each covariable by the use of arithmetic operators (subtraction). The algebraic analysis of maps (spatial variables, response variable and covariables), represented by pixels, results a new map where the values in each geographical position was the result of subtraction (in our case) of the values of the variables associated with the geographical position.

The term “map algebra” was established by Dana Tomlin in the early 1980s [32] with the development of the “Map Analysis Package GIS”. Map algebra provides tools to perform spatial analysis operations and is based on a matrix algebra, which refers to the algebraic manipulation of matrices (as maps in raster data structures). Spatial data were analyzed in a GIS platform using ArcGis 9.3 software (Environmental Systems Research Institute, Redlands, CA, USA).

### Statistical analysis

For the analysis of the proposed hypothesis (small wild mammal lower richness is associated with the increase of prevalence of *T. cruzi* infected dogs), we used the GLMs (Generalized Linear Models) with a Poisson link function [33]. For the response variable we used the infection of the dogs (based on serological assays – IFAT and ELISA, as described above). The following covariables were included: (1) Species richness of small wild mammals collected (DS): The richness was calculated as being the number of species captured in each area; (2) Abundance of small wild mammals collected (NM): The abundance for each localities was based on: n = total number of mammals captured. In the present model, aiming to evaluate the influence of both parameters (normally associated in ecological studies), these two covariables (DS*NM) were considered together and estimated by the “manual” selection method; (3) Prevalence of small wild mammals with positive *T. cruzi* parasitological assays (THC): That included (i) the finding of flagellates with typical *T. cruzi* morphology in fresh blood examination and (ii) the isolation and characterization of *T. cruzi* from blood in axenic medium – hemoculture; (4) Prevalence of small wild mammals with positive *T. cruzi* serological assay: based on the detection of specific anti-*T. cruzi* antibodies in the IFAT. The criterion of comparison between the models was based on the Akaike Information Criterion (AIC) and residual deviance [34], [35] to determine which model fits best considering the level of significance (p<0.05). For the analysis of normality, the Shapiro-Wilk normality test was performed. Each model is specified as a combination of covariables that can influence the probabilities of dogs becoming infected. The comparison between dogs from Chagas disease-outbreak and non-outbreak areas were calculated using 2×2 contingency tables along with a Chi-squared test. Each dog was considered as one independent event, even when living in the same house. Both analyses were performed using the software R (Version 2.11.1) [36].

### Owner consent and protocol of ethical treatment of animals

All wild animal manipulation procedures were performed in accordance with the COBEA (Brazilian College of Animal Experimentation) following the guidelines of the Animal Ethics Committee (CEUA) protocol of FIOCRUZ (Oswaldo Cruz Institute Foundation), Ministry of Health, Brazil. All field workers who manipulated animals directly were adequately dressed with protective equipment, following protocols previously approved by the CEUA-FIOCRUZ Committees of Biosafety and of Bioethics (licenses: P0007-99; P0179-03; L0015-07; P0292/06). The wild animal captures were licensed by the Brazilian Institute of Environment and Renewable Natural Resources (IBAMA) licenses 068-2005 and 225-2006 (IBAMA/CGFAU/LIC). In all cases, consent from the dog owners was obtained. In addition, the owners also helped handle the animals during sampling to avoid incidents. A canine standard questionnaire was applied. For each dog, the questionnaire included name, sex, age, size, color and main phenotypic features, birthplace, age at which the pet entered the house, the dog's main function and movement areas.

## Results

### Parasitological and molecular characterization

**Figure 2:**
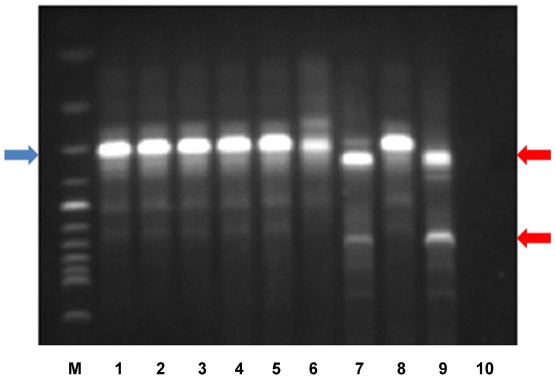
*Trypanosoma* sp. characterization in blood samples from dogs from Pará State. Parasite identification targeting the variable region of minicircle kDNA, **Lanes 1–5** DNA extracted from serum samples of dogs designated LBT1818, LBT1819, LBT1820, LBT1821 and LBT1822, respectively; **lane 6**: FNS258; **lane 7** LBT1831; **lane 8** *T. cruzi* positive control; **lane 9** *T. rangeli* positive control; **lane 10** negative control; **lane M**: 100-bp DNA ladder. **Red arrows**: 760 bp fragment and fragments varying in size from 300 to 450 bp derived from *T. rangeli* minicircles; **Blue arrow**: 330-bp fragment derived from *T. cruzi* minicircles.

*Trypanosoma cruzi* infection in dogs was sub-patent in the majority of the cases. Only five dogs from Monte Alegre displayed trypomastigote forms in fresh blood preparations (Table 1). These dogs with patent parasitemia displayed severe clinical symptoms (fever, pale mucous membranes, generalized edema, rigid abdomen and splenomegaly), and the disease was fatal for two of them. Hemocultures performed in the three dogs surviving two months after the first blood collection were negative; these dogs produced Chagas negative hemocultures 3 months after the first blood collection. Positive hemocultures were detected only in two dogs, one from Abaetetuba and one from Curralinho. From one of these two dogs, it was possible to isolate the *T. cruzi* on two occasions after an interval of seven months (Abaetetuba). Molecular characterization was performed on these isolates and on five serum samples of dogs from Monte Alegre. *T. cruzi* k-DNA was amplified from these six dogs, from both serum and culture samples, with the exception of one dog from Curralinho with *Trypanosoma rangeli* (Figure 2 and Table 1).

### Serological data

**Figure 3:**
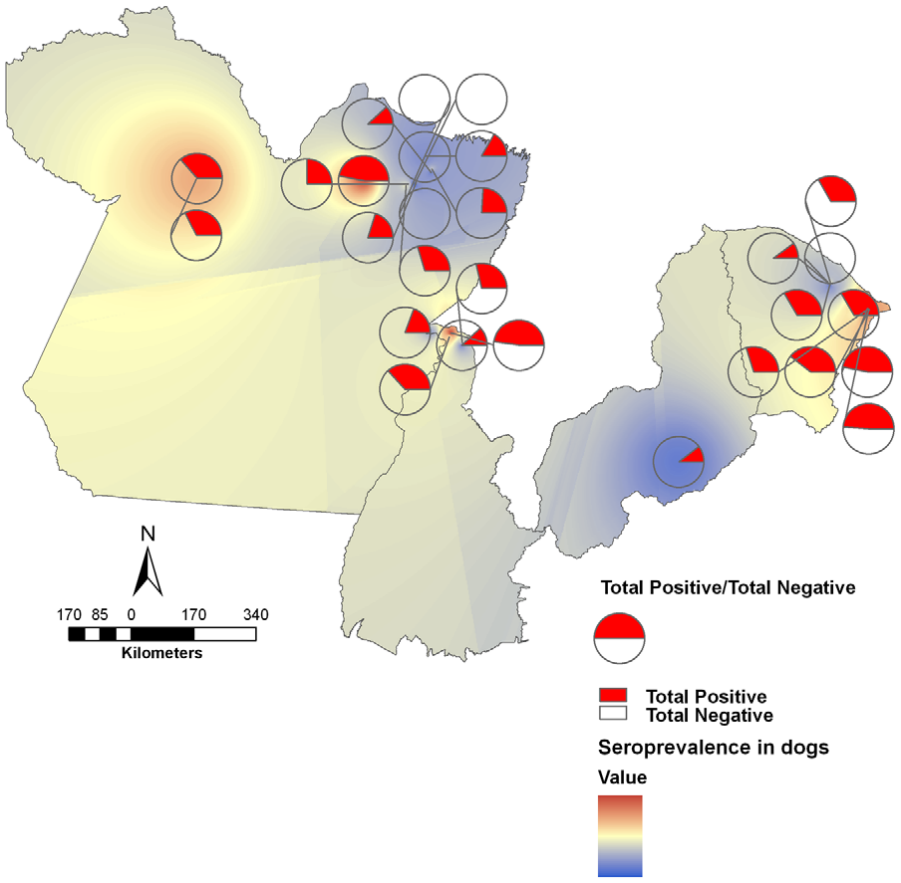
Mapping of distribution of *Trypanosoma cruzi* infection in dogs (municipalities). Distribution of seroprevalence in dogs by the interpolation maps method (Inverse Distance Weighted - IDW): Total positive/Total tested.

Overall, the distribution of *T. cruzi* infection in dogs was not homogeneous among houses, localities, municipalities and/or biomes (Figure 3), as shown by the high variation in *T. cruzi* prevalence in dogs in the different municipalities (11–89%, Table 1). Within the same biome, municipalities with high and low *T. cruzi* seroprevalence in dogs were observed. In the Jaguaruana and João Costa municipalities located in the Caatinga biome, dogs displayed 71% and 11% seropositivity, respectively. Similar differences in dog seroprevalence were also observed in distinct localities in the Amazon biome (22–89%). Even within the same municipality, seroprevalence in dogs was not homogeneous. In Abaetetuba (Amazonia biome), the seroprevalence in dogs ranged from 0 to 42% according to the locality. In the Pantanal, where Chagas disease is not reported, all dogs serologically examined were negative for *T. cruzi* infection (n = 39) (Table 1). Each dog was considered as one independent event, even when living in the same house due do the fact that different seroprevalence rates could be observed in dogs of one same house. The not homogeneous distribution of *T. cruzi* seroprevalence in dogs of one single house reflect that these dogs have not been equally exposed to *T. cruzi* infection.

### Co-infection with *Leishmania infantum*

Co-infection was observed in 17% and 16% of sera from dogs examined in the Caatinga and Amazon biomes, respectively, as evaluated by the IFAT and/or Quick Test for Diagnosis of Visceral Leishmaniasis (CVL) (TR DPP®, Bio-Manguinhos, FIOCRUZ, Rio de Janeiro, RJ, Brazil), indicating co-infection by both parasite species.

### Geospatial analysis

**Figure 4:**
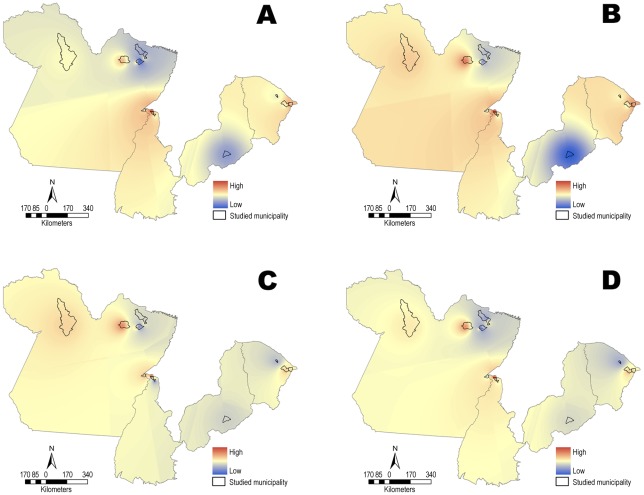
Mapping of lower richness of wild mammal species. A geospatial analysis by the map algebra of the association of *T. cruzi* infection in dogs (response variable) with covariables: (**A**) species richness (DS) of small wild mammals; (**B**) abundance (NM) of small wild mammals; (**C**) serological prevalence (IFAT) of small wild mammals; (**D**) parasitological prevalence (THC) of small wild mammals.

An analysis of the maps generated using the interpolation method indicated an inverse distribution correlation among *T. cruzi* infection in dogs and a decrease in the richness and abundance of small wild mammal species. This spatial correlation evidence was confirmed by map algebra and demonstrated that among the response variable and covariables there is also an inverse correlation, which indicated that in areas with greater richness and abundance of small mammal species, dogs were less prone to be infected with *T. cruzi*. Since the resulting map algebra was subtracted from inverse correlation with two variables our result shows a distribution that is not homogeneous. A more indirect indication was given by the rates of parasitological and serological *T. cruzi* prevalence in dogs (Figure 4A–D).

### Statistical analysis

Statistical analysis confirmed the generated maps and demonstrated that the covariables DS (Species Richness) and THC (parasitological prevalence) influence the average response variable (*T. cruzi* infection in dogs), polygons of the studied municipalities. The estimated DS (−0.095596) indicates that in areas that present greater mammal biodiversity, dogs are less prone to infection by *T. cruzi*. The estimated TCH (0.009066) indicated that in areas that present higher parasitological prevalence of infection in the small wild mammals, dogs are more exposed to the *T. cruzi* infection. The estimated rate of *T. cruzi* infection in dogs was 0.909 (CI_95%_ 0.870–0.949) for DS and 1.009 (CI_95%_ 1.004–1.014) for TCH. The analysis of residuals versus fitted values indicated that the behavior of the variance of residuals and homoscedasticity presented random residuals. For confirmation of the normality of the data, the Shapiro-Wilk test was performed (W = 0.9814), *P* = 0.8714 at 5%. We found a significant difference in the *T. cruzi* infection rate between dogs sampled from areas that suffered a Chagas disease outbreak compared to dogs from non-outbreak areas (28/103 versus 217/546, Chi-squared 5.81, *P* = 0.01). In other words, this probability would rise 0.5 points in areas with Chagas disease outbreaks.

## Discussion

The sustainability of successful control of Chagas disease requires a more accurate knowledge of the environmental factors that underlie the transmission cycle of this parasite in the wild, mainly, if there are still unknown and undetermined aspects of the current epidemiology of this trypanosomiasis. This demands multidisciplinary and complex studies, as *Trypanosoma cruzi* is a multihost parasite that displays a huge intraspecific heterogeneity and a complex transmission cycle that may exhibit local peculiarities. Oral transmission of *T. cruzi* to humans was reported as sporadic until 2004, but in the following years, this epidemiological profile of transmission became increasingly important in the epidemiology of Chagas disease, particularly in the Amazon region [4]–[6]. Outside this region, oral transmission has also been responsible for recent outbreaks of ACD in several Brazilian states, mainly in the North [2], [3]. Such outbreaks have also been reported in other Latin American countries [37], [38].

Our results indicate that infection by *T. cruzi* in dogs is not homogeneous but focal, as demonstrated by the differences in seroprevalence among close localities; these differences may be due to landscape features. The seroprevalence observed in dogs could be associated with their proximity to forest and rural areas and with the loss of richness and abundance and rates of infection of the small wild mammal fauna. One aspect that distinguishes the present and previous data of our group from other studies is the scarcity of the number of dogs that displayed positive hemocultures [17]–[19]. In fact, dogs in Brazil are apparently only rarely involved in the amplification of *T. cruzi* and seem to play a minor role in the dispersion of the parasite. Even the dogs from Monte Alegre/PA seem not be of epidemiological importance because hemocultures were negative 3 months after the detection of *T. cruzi* in their blood smears.

The importance of a host species as a reservoir of a vector-borne parasite mainly depends on its prevalence of infection, capacity to infect the vectors, and the rate of host-vector contact [39]. A possible consequence of a local simplification of the small wild mammal fauna, where generalist mammals are favored at the expense of specialist species, is an increase in the rate of infection among the remnant mammalian fauna when the selected species are competent reservoirs of *T. cruzi*. As a result, the parasite population increases in the area, favoring vector infection and exposure of dogs to parasites, as reflected by their seroprevalence. This scenario suggests that the assessment of potential disease risk factors requires detailed knowledge of local, site-specific conditions. The small wild mammalian fauna diversity plays an important role in the profile of the enzootic infection patterns in a given area, as shown by the high transmission focus described in a previous study [22].

Overall, despite many remaining questions, the current evidence indicates that preserving intact ecosystems and their endemic biodiversity should generally reduce the prevalence of infectious diseases [7], [8].

The determination of the spatial distribution of the elements that compose the epidemiological chain of a parasitic disease is of pivotal importance for the determination of trends and risk evaluation. Moreover, it is worth mentioning that the attempts to control a given multihost parasite based on the control of one single vector or host species will always be insufficient because parasite transmission very rarely relies on a single system. The simplification of the mammalian host diversity, associated with an increase in the abundance of competent reservoir host species as described here is certainly one of the risk factors involved in the reemergence of Chagas disease [9]. Reduced disease risk with increasing host diversity is especially likely when pathogen transmission is frequency-dependent, and when pathogen transmission is greater within a species than between species, particularly when the most competent hosts are also relatively abundant and widespread [7].

Piauí and Ceará display similar patterns regarding the presence of a high density of naturally *T. cruzi* infected Triatominae, which are the main vectors in both regions. Our results demonstrate a high prevalence of *T. cruzi* infection in dogs from the Caatinga, as described in previous studies from our group [9], [22]. Despite this high prevalence, no new Chagas disease cases of vectorial transmission have been observed there in the last decade [13], [26]. This may reflect the effectiveness of the already long-lasting epidemiological surveillance campaigns exerted in these areas despite their lack of regularity. Local people are aware of the risk of disease and adopt local measures to avoid infection risk. Further, although dogs were exposed to the *T. cruzi* transmission cycle and are hosts of the parasite, they do not display high parasitemia (i.e., had negative hemocultures) and are therefore not involved in the amplification of parasite populations, so consequently, the potential for transmission from these dogs to the vector is low.

The high prevalence of seropositive dogs in the Amazon region can be attributed to the elevated rate of contact among these domestic animals and the wild environment and because the houses are practically located inside wild forest areas. In these areas, it is difficult to delimit the of peridomestic and wild areas, and many local inhabitants and dogs are involved in hunting activities. Empirical evidence indicates that habitat fragmentation can increase or decrease disease prevalence (and also *T. cruzi* infection among wild small mammals) within a host species, depending on the specific biology of the host–parasite relationship [28], [40]. Another important factor that should be taken into account is the importance of the definition of risk area based on the characteristics of the micro-regional management of domestic animals that are sometimes reared in semi-extensive ways. In this case, these animals are more exposed to the wild cycle of transmission and this is reflected by a high prevalence of infection. The presence of seropositive dogs in strictly domiciled habitats, as observed in a previous study in Navegantes, in the state of Santa Catarina, indicates, for example, the presence of a transmission cycle very close to the animal's home [2]. Moreover, the high prevalence of infection in domestic mammals reared in a semi-extensive way (such as pigs from Cachoeira do Arari/PA) indicates that transmission is occurring farther from homes but within the areas of interface between the peridomestic and wild environments [16].

Surveillance for canine Chagas disease should be a useful tool for the design of suitable epidemiological control programs in areas where sylvatic triatomines are responsible for human infection, as in many rural endemic areas [17].

The geospatial analysis approach involving interpolation and the map algebra method are a powerful tool in the study of the association between lower richness and areas with high transmission rates in small wild mammals and the risk of exposure of dogs to *T. cruzi* infection. Dogs are important sentinels and efficient indicators of areas at risk for Chagas disease outbreaks, lower richness in wild mammalian fauna diversity and selection of suitable *T. cruzi* reservoir hosts.

Therefore, the monitoring of domestic animals can and should be used as a first measure in the diagnosis of areas with elevated risk of *T. cruzi* transmission. Dogs, in particular, are easy to handle and have a generally accessible traceability. The collection of blood from these hosts and serologic testing (the sending of material to a central diagnostic institute) does not require great cost and infrastructure. Moreover, blood samples can be easily obtained in areas where dogs are routinely collected and tested for *Leishmania* sp. Or the anti-rabies vaccination campaigns can be used to collect blood from a representative sample of dogs in a given area. The presence of seropositive dogs reflects exposure to *T. cruzi* and points to the transmission of the parasite in areas where these animals roam. Once this measure is implemented, we should have an efficient indicator of areas at risk for human Chagas disease that require particular epidemiological investigation, implementation of control measures and health education.

## Supporting Information

Figure S1 **Mapping of distribution of parasitological prevalence, richness and abundance of small wild mammals.** Geospatial analysis showed that the lower richness of the mammal fauna (richness and abundance) was associated with higher parasitemia in small wild mammals and higher exposition of dogs to infection.
(TIF)

Table S1 **Prevalence of infection by** ***Trypanosoma cruzi*** **in small wild mammals.** Caatinga, Amazon Forest and Pantanal. Richness indicates the number of species captured in each area; Prevalence of small wild mammals with positive *T. cruzi* parasitological assays includes mammals that displayed: flagellates with typical *T. cruzi* morphology in fresh blood examination and/or positive hemoculture, *i.e.*, isolation and characterization of *T. cruzi* from blood in axenic medium; Prevalence of small wild mammals with positive *T. cruzi* serological assay was based on the detection of specific anti-*T. cruzi* antibodies in the IFAT.
(DOC)
