# Supplementary figures and images for: Lower Richness of Small Wild Mammal Species and Chagas Disease Risk
Source: PLoS Negl Trop Dis. 2012 May 15;6(5):e1647. doi: 10.1371/journal.pntd.0001647 (PMC3352825; doi:10.1371/journal.pntd.0001647)

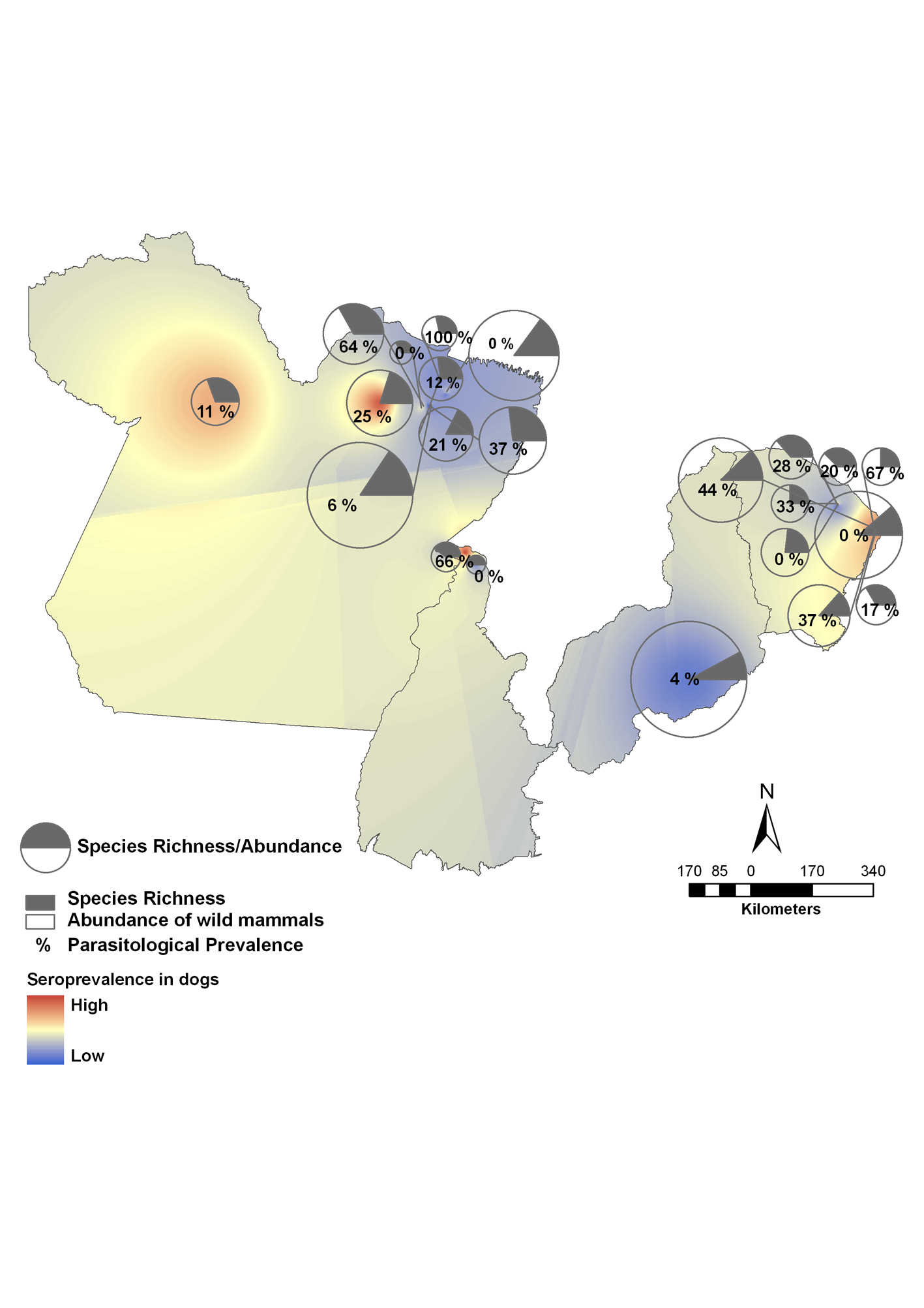

Supplement: Figure S1 — Mapping of distribution of parasitological prevalence, richness and abundance of small wild mammals. Geospatial analysis showed that the lower richness of the mammal fauna (richness and abundance) was associated with higher parasitemia in small wild mammals and higher exposition of dogs to infection. (TIF) [file pntd.0001647.s001.tif]
