# Supplementary material for: Lower Richness of Small Wild Mammal Species and Chagas Disease Risk
Source: PLoS Negl Trop Dis. 2012 May 15;6(5):e1647. doi: 10.1371/journal.pntd.0001647 (PMC3352825; doi:10.1371/journal.pntd.0001647)
Supplement: Table S1 — Prevalence of infection by Trypanosoma cruzi in small wild mammals. Caatinga, Amazon Forest and Pantanal. Richness indicates the number of species captured in each area; Prevalence of small wild mammals with positive T. cruzi parasitological assays includes mammals that displayed: flagellates with typical T. cruzi morphology in fresh blood examination and/or positive hemoculture, i.e., isolation and characterization of T. cruzi from blood in axenic medium; Prevalence of small wild mammals with positive T. cruzi serological assay was based on the detection of specific anti-T. cruzi antibodies in the IFAT. (DOC) [file pntd.0001647.s002.doc]

**Table S1: Prevalence of infection by *Trypanosoma cruzi* in small wild mammals**: Caatinga, Amazon Forest and Pantanal.

|  |  |  | **Small Wild Mammals** | | |
| --- | --- | --- | --- | --- | --- |
| **Biome/State** | **Municipaltities** | **Localities** | **Species Richness** | **IFAT +/Total (%)** | **Hemoculture +/Total (%)** |
| **Caatinga** | Jaguaruana | Caatinguinha | 5 | 20/36 (55) | 16/36 (44) |
| Ceará |  | Córrego das Melancias | 3 | 14/19 (77) | 7/19 (37) |
|  |  | Dió | 3 | 2/6 (33) | 1/6 (17) |
|  |  | Perímetro Irrigado | 2 | 2/2 (100) | 1/2 (50) |
|  |  | Figueiredo do Bruno/do Ivan | 2 | 5/6 (83) | 4/6 (67) |
|  |  |  |  |  |  |
|  | Redenção | Salobro | 3 | 7/10 (70) | 0/10 |
|  |  | Alto Cassiano | 4 | 5/7 (71) | 2/7 (28) |
|  |  | Manoel Dias | 2 | 3/6 (50) | 2/6 (33) |
|  |  | Sítio Outeiro | 3 | 4/5 (80) | 1/5 (20) |
|  |  |  |  |  |  |
|  | Russas | Cipó | 5 | 8/39 (20) | 0/39 |
|  |  |  |  |  |  |
| Piauí | João Costa |  | 6 | 12/71 (17) | 3/71 (4) |
|  |  |  |  |  |  |
| **Amazon Forest** |  |  |  |  |  |
| Tocantins | Augustinópolis | 2000 | 0 | - | - |
|  |  | São Roque | 2 | 3/3 (100) | 2/3 (66) |
|  |  |  |  |  |  |
|  | Esperantina | São Francisco | 0 | - | - |
|  |  |  |  |  |  |
|  | Axixá do Tocantins | Lagoa de São Salvador | 0 | - | - |
|  |  | Piquizeiro | 2 | 1/1 (100) | 0/1 |
|  |  |  |  |  |  |
|  |  |  |  |  |  |
| Pará | Abaetetuba | Ajuaí | 6 | 8/14 (57) | 9/14 (64) |
|  |  | Genipauba | 6 | 12/19 (63) | 7/19 (37) |
|  |  | Panacaueira | 1 | 0/2 | 0/2 |
|  |  | Urban Area | 8 | 15/54 (28) | 3/54 (6) |
|  |  |  |  |  |  |
|  | Belém | Jurunas | 0 | - | - |
|  |  | Val de Cans | 6 | 24/40 (60) | 0/41 |
|  |  |  |  |  |  |
|  | Cachoeira do Arari | Aranaí | 2 | 4/5 (80) | 5/5 (100) |
|  |  | Furinho | 3 | 3/14 (21) | 3/14 (21) |
|  |  | Mata Fome | 3 | 3/8 (37) | 1/8 (12) |
|  |  | Sede Furo Grande | 0 | - | - |
|  |  |  |  |  |  |
|  | Curralinho | São José da Povoação | 5 | 6/20 (30) | 5/20 (25) |
|  | Monte Alegre | setor 11 | 4 | 2/9 (22) | 1/9 (11) |
|  |  |  |  |  |  |
| **Pantanal** |  |  |  |  |  |
| Mato Grosso do Sul | Corumbá | Farms | 17 | 31/95 (32) | 35/221 (16) |
|  |  |  |  | **81/206 (39)** | **42/206 (20)** |

**Legend:**

Richness indicates the number of species captured in each area; Prevalence of small wild mammals with positive *T. cruzi* parasitological assays includes mammals that displayed: flagellates with typical *T. cruzi* morphology in fresh blood examination and/or positive hemoculture, *i.e.*, isolation and characterization of *T. cruzi* from blood in axenic medium; Prevalence of small wild mammals with positive *T. cruzi* serological assay was based on the detection of specific anti-*T. cruzi* antibodies in the IFAT.

**Footnotes:**

**IFAT -** Indirect Immunofluorescence Antibody Test

1-Data published in [22]

2-Data published in [2]
